# Dynamic Enhancement for Dual Active Bridge Converter with a Deadbeat Current Controller

**DOI:** 10.3390/mi13122048

**Published:** 2022-11-23

**Authors:** Chengfu Tian, Shusheng Wei, Jiayu Xie, Tainming Bai

**Affiliations:** 1 Unit 91977 of the Chinese People’s Liberation Army, Beijing 100036, China; 2 School of Automotive Engineering, Wuhan University of Technology, Wuhan 430070, China

**Keywords:** dual active bridge, deadbeat controller, load feedforward

## Abstract

This paper investigates the deadbeat current controllers for isolated bidirectional dual-active-bridge dc-dc converter (IBDC), including the peak current mode (PCM) and middle current mode (MCM). The controller uses an enhanced single phase shift (ESPS) modulation method by exploiting pulse width as an extra control variable in addition to phase shift ratio. The control variables for PCM controllers are derived in detail and the two different current controllers are compared. A double-closed-loop control method is then employed, which could directly control the high-frequency inductor current and eliminate the transient DC current bias of the transformer. Furthermore, load feedforward was introduced to further enhance the dynamic of the converter. With the proposed control method, the settling time could be reduced within several PWM cycles during load disturbance without transient DC current bias. A 5 kW IBDC converter prototype was built and the settling time of 6 PWM cycles during load change with voltage regulation mode was achieved, which verifies the superior dynamic performance of the control method.

## 1. Introduction

The isolated bi-directional dual-active-bridge dc-dc converter (IBDC) has been a hot topic in recent years due to its simple structure, high efficiency and ultrafast response [1]. The transient DC current offset of the transformer and the inductor, which might saturate the transformer and increase the system’s current stress during the abrupt load change, has attracted people’s attention. Different dynamic modulation methods have been proposed to solve the problem [2,3,4,5]. Additionally, to increase the dynamics of IBDCs, the current mode controller could be a competitive alternative. It also has other inherent benefits including over-current protection, elimination of transient DC current offset and easy implementation of current sharing between multiple IBDCs [6].

Digital predictive current controllers based on conventional single phase shift (CSPS) modulation was proposed in [7,8], where the phase shift ratio was used to control the transformer current. In [7], the average current calculated by an analog integrator of the DC bus current was used as the feedback signal, which can achieve fast dynamic performance. However, transient DC current offset occurs during the sudden change in phase shift ratio for CSPS modulation.

**Figure 1:**
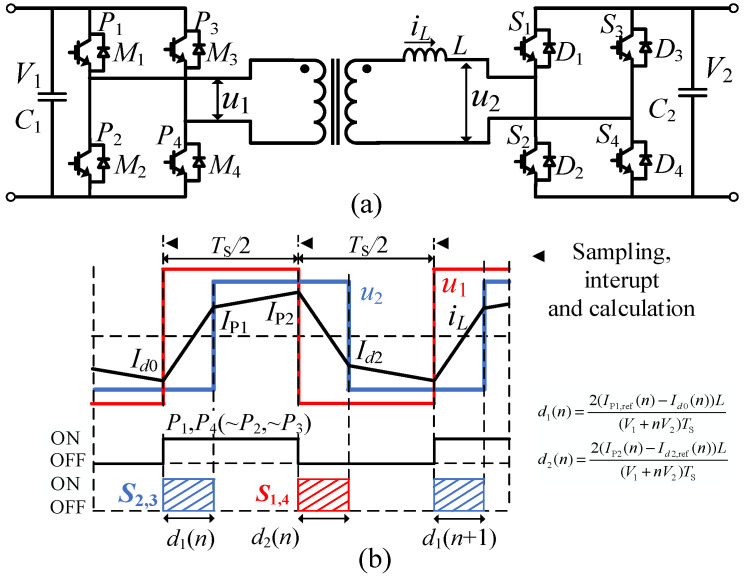
Principle of the PDCM controller. (**a**) IBDC, (**b**) waveforms.

The predictive duty cycle mode (PDCM) controller, shown in Figure 1b, was proposed in [6] to eliminate the transient DC current offset, which was applied in [8]. The drive signals of the primary side are fixed. The transformer current needs to be oversampled, and duty cycles d1 for S2,3 and d2 for S1,4 are calculated in turn in every half cycle. Another limitation of this method is that the controller only works in the ZVS range (IP1 > 0) shown in Figure 1b and may lose effectiveness when IP1 < 0. 

A deadbeat current controller based on the middle current and enhanced PWM modulation was proposed in [9]. However, regulation of the output voltage and current was not introduced, which is more important in real application. To overcome the drawbacks, this paper investigated the deadbeat current controllers, including the peak current mode (PCM) and middle current mode (MCM). Based on the controllers, a double-closed-loop control method with load feedforward was introduced. Furthermore, a 5 kW IBDC converter prototype was built, and the settling time of 6 PWM cycles during load change could be achieved, which validates its superior dynamic performance.

## 2. Deadbeat Peak Current Mode Controller 

### 2.1. Basic Model of IBDC for SPS Modulation

**Figure 2:**
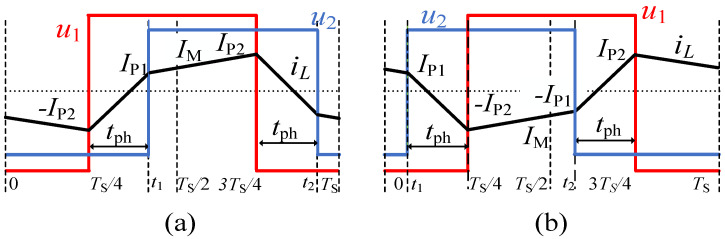
Waveforms of IBDC at steady state for CSPS modulation: (**a**) forward power transmission (*P* > 0), (**b**) reverse power transmission (*P* < 0).

The basic model of SPS modulation-based IBDC is presented prior to introducing the proposed current controller. Figure 2 illustrates the theoretical waveforms of the IBDC using the SPS modulation method when converter voltage gain k≥1, where *k* = *V*_1_/(n*V*_2_) and n is the turn ratio of the transformer. The waveforms are symmetrical for the same transmission power of two opposite directions.

The symbols in Figure 2 are defined as follows: TS is the switching cycle, f is the switching frequency, *D* is the phase shift ratio and tph is the shifted time. *D* ≥ 0 (*t*_ph_ ≥ 0) stands for *P* ≥ 0 and *D* < 0 (*t*_ph_ < 0) for *P* < 0. *I*_P1_ and *I*_P2_ are the two “switching currents” and *I*_P2_ is the peak current when *k* > 1. The middle current IM, defined as the instantaneous current at *T*_S_/2, is taken into consideration instead of the average current, which equals zero in one cycle. The basic equations for the IBDC are derived as follows:

(1) $$\left\{ \begin{array}{l} P=\frac{V_{1}{}^{2}}{2fLk}D(1-\left|D\right|)\;,\;t_{\mathrm{ph}}=\frac{DT_{S}}{2}\\ I_{P1}=\frac{V_{1}(2k\left|D\right|-k+1)}{4fLk},\;I_{P2}=\frac{V_{1}(2\left|D\right|+k-1)}{4fLk},\;I_{M}=\frac{V_{1}D}{2fLk} \end{array}\right.$$

The relationships among the variables *P*, *I*_P1_, *I*_P2_, *I*_M_, *D* and *t*_ph_ at steady state can then be derived. Therefore, for a given value of one variable, other variables can be calculated.

### 2.2. Peak Current Mode Controller

**Figure 3:**
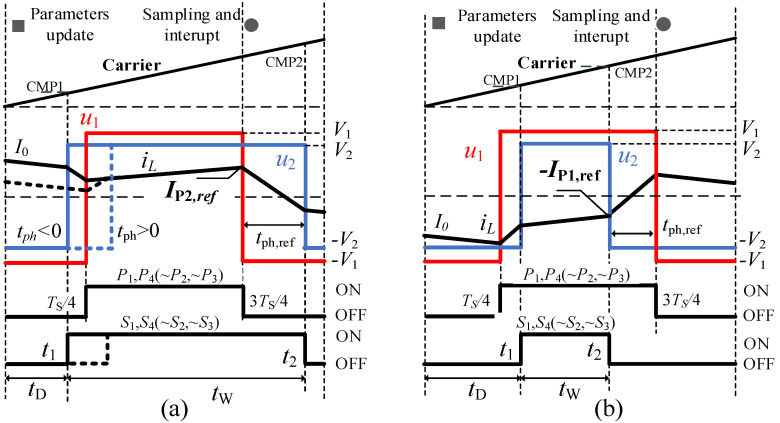
Transient waveforms of PCM controller: (**a**) forward power transmission (*P*_ref_ > 0), (**b**) reverse power transmission (*P*_ref_ < 0).

Peak current mode (PCM) controllers are introduced in this section. Figure 3a,b show the transient waveforms of a PCM current controller in one cycle for forward and reverse power transmission, respectively. *P*_1_, *P*_2_, *P*_3_ and *P*_4_ are the drive signals for the primary side and *S*_1_, *S*_2_, *S*_3_ and *S*_4_ are the drive signals for the secondary side. The variable *t*_ph,ref_ is shifted-time at the steady state for the given *I*_P2,ref_ which can be derived from (1). A sawtooth carrier with the same frequency of the converter was utilized to generate the reference signals. The “switching on” moment *t*_1_ and “switching off” moment *t*_2_ should meet the constrains as: 0 < *t*_1_ < *T*_S_/2 and 3*T*_S_/4 < *t*_2_ < *T*_S_. The variables *t*_D_ and *t*_W_ are defined as “delay time” and “width time”, respectively. *P*_ref_ is the power reference, and *I*_P2,ref_ and *I*_P1,ref_ are the references for the corresponding “switching currents”, respectively.

As shown in Figure 3a, there are two cases according to the initial current *I*_0_ and reference current *I*_p2,ref_: *u*_2_ leads *u*_1_ (*t*_ph_ < 0) for the solid line waveforms and *u*_2_ lags *u*_1_ (*t*_ph_ > 0) for dotted line waveforms. For the sake of brevity, the superposition principle was used to derivate the inductor current when calculating *t*_D_ and *t*_W._

For forward power transmission, the requirement was imposed that *I*_P2_ = *I*_P2,ref_ and *t*_ph,ref_ > 0. According to the superposition principle, the inductor current ripple $\Delta I_{L}$ during 0 and 3*T*_S_/4 can be calculated by adding up the two ripple currents as follows:

(2) $$\left\{ \begin{array}{l} \Delta I_{L}=\Delta I_{L,u_{1}}+\Delta I_{L,u_{2}}\;\\ \Delta I_{L,u_{1}}=\frac{-V_{1}}{L}\cdot \frac{T_{S}}{4}+\frac{-V_{1}}{L}\cdot \frac{T_{S}}{2}\;,\;\Delta I_{L,u_{2}}=\frac{V_{2}}{L}\cdot t_{D}+\frac{-V_{2}}{L}\cdot (\frac{3T_{S}}{4}-t_{D})\\ I_{P2}=I_{0}+\Delta I_{L}=I_{P2,\mathrm{ref}} \end{array}\right.$$

where $\Delta I_{L,u_{1}}$ and $\Delta I_{L,u_{2}}$ are current ripples generated by the two dependent voltage source *u*_1_ and *u*_2_, respectively. *t*_D_ is then derived as:

(3) $$t_{D}=\frac{(I_{P2,\mathrm{ref}}-I_{0})Lk}{2V_{1}}+\frac{3-k}{8f}$$

Furthermore, *t*_W_ is derived as:

(4) $$t_{W}=\frac{3T_{S}}{4}-t_{D}+t_{\mathrm{ph},\mathrm{ref}}$$

**Figure 4:**
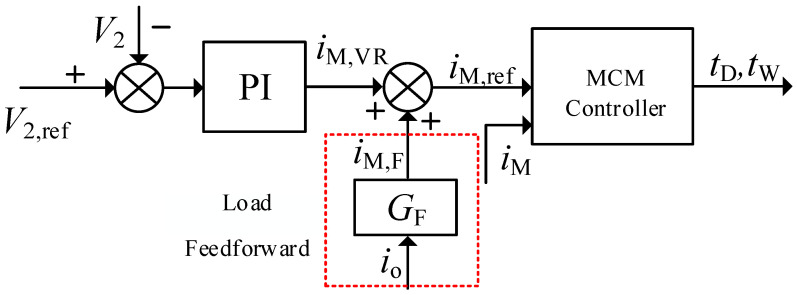
Voltage control scheme based on the MCM-ESPS controller.

With regard to reverse power transmission, the switching current at *t*_2_ is set to be—*I*_P1,ref_ and *t*_ph,ref_ < 0 as shown in Figure 4. Similar to forward power transmission, *t*_D_ and *t*_W_ can be obtained. Thus, the equations for the PCM controller are written as:

(5) $$\left\{ \begin{array}{l} t_{D}=\frac{kL(I_{P2,\mathrm{ref}}-I_{0})}{2V_{1}}+\frac{3-k}{8f};\;t_{W}=\frac{kL(I_{P2,\mathrm{ref}}+I_{0})}{2V_{1}}-\frac{k-5}{8f},\;P_{\mathrm{ref}}\geq 0\\ t_{D}=-\frac{kL(I_{P2,\mathrm{ref}}+I_{0})}{2V_{1}}+\frac{k+1}{8f};\;t_{W}=-\frac{kL(I_{P2,\mathrm{ref}}-I_{0})}{2V_{1}}+\frac{k+3}{8f},\;P_{\mathrm{ref}}<0 \end{array}\right.$$

As in the aforementioned Equations (6) and (7), initial current *I*_0_ is sampled to calculate the *t*_D_ and *t*_W_. However, a one-cycle delay exists between the sampling instant and control update due to the algorithm implementation of the digital processor. *I*_P2_ for the PCM controller is sampled at 3*T*_S_/4, and DSP interrupt occurs to calculate the new parameters shown in Figure 3. *t*_D_ and *t*_W_ update at the beginning of the next cycle. Assuming DC bus voltage *V*_1_ and *V*_2_ are constant in two adjacent periods, the relationships between the *I*_P2_(*n* − 1), *I*_M_(*n* − 1) in the (*n* − 1)th cycle and the initial current in the *n*th switching cycle *I*_0_(*n*) could be derived as:

(6) $$I_{0}(n)=\left\{ \begin{array}{l} I_{P2}(n-1)-\frac{2V_{1}(k-1)}{Lk}(t_{D}(n-1)+t_{W}(n-1))+\frac{V_{1}(7-k)}{4fLk}\;,\;P_{\mathrm{ref}}(n-1)\geq 0\\ I_{P2}(n-1)-\frac{V_{1}(k-1)}{4fLk},\;P_{\mathrm{ref}}(n-1)<0 \end{array}\right.$$

According to the power transmission directions in two adjacent cycles, four situations are considered for the PCM controller: case 1 when *P*_ref_(*n* − 1) ≥ 0 and *P*_ref_(*n*) ≥ 0; case 2 when *P*_ref_(*n* − 1) < 0 and *P*_ref_(*n*) ≥ 0; case 3 when *P*_ref_(*n* − 1) ≥ 0 and *P*_ref_(*n*) < 0; and case 4 when *P*_ref_(*n* − 1) < 0 and P_ref_(*n*) < 0. Combining (6)–(9), the control variables *t*_D_ and *t*_W_ with delay compensation can be derived as shown in Table 1. With the control variables in Table 1, the inductor peak current could be tracked to the reference in two cycles, which is consistent with the idea of the deadbeat control in ref [10].

## 3. Double-Closed-Loop Control with Load Feedforward

In practice, instead of the high-frequency inductor current, the DC voltage, current or power should always be regulated. In this section, the voltage mode control strategy based on the MCM-ESPS controller is introduced.

Figure 4 shows the output voltage control scheme based on the deadbeat current controller, where two control loops are involved. The load feedforward control could substantially increase the system dynamic [11,12]. In order to improve the stability of output voltage under load disturbance, load feedforward under double-closed-loop control is presented. As shown in Figure 4, the feedforward current *i*_M,F_ corresponding to the load was superimposed on the current reference *i*_M,VR_, which is the output of the outer voltage loop, to form the final current reference value *i*_M,ref_.

The relationships of the middle current were derived as:

(7) $$I_{M}=\left\{ \begin{array}{l} \frac{V_{1}}{2fLk}(\frac{1}{2}-\sqrt{\frac{1}{4}-\frac{2fLkP}{V_{1}{}^{2}}})\;,\;P\geq 0\\ -\frac{V_{1}}{2fLk}(\frac{1}{2}-\sqrt{\frac{1}{4}+\frac{2fLkP}{V_{1}{}^{2}}})\;,\;P<0 \end{array}\right.$$

Without considering the power loss of the converter, we could obtain:

(8) $$P=V_{2}i_{o}$$

Thus, the relationship between the middle current *I*_M_ and the load current *i*_o_ could be expressed as (9) and (10):

(9) $$I_{M}=\left\{ \begin{array}{l} \frac{V_{1}}{2fLk}(\frac{1}{2}-\sqrt{\frac{1}{4}-\frac{2fLi_{o}}{V_{1}}})\;,\;i_{o}\geq 0\\ -\frac{V_{1}}{2fLk}(\frac{1}{2}-\sqrt{\frac{1}{4}+\frac{2fLi_{o}}{V_{1}}})\;,\;i_{o}<0 \end{array}\right.$$

(10) $$i_{o}=NI_{M}(1-\frac{2fLkI_{M}}{V_{1}})$$

**Figure 5:**
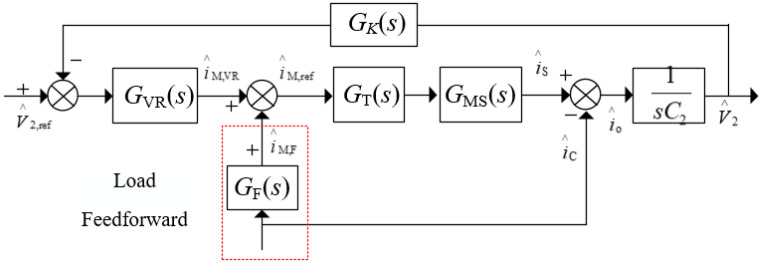
Small signal model of an IBDC with the double-closed-loop control strategy.

The small signal model of the system, as shown in Figure 5, can be obtained from the control block diagram in Figure 4, where *i*_S_ is the average output current of an H bridge in a single period and *G*_o_(*s*) is the transfer function of capacitance voltage and capacitance current, denoted as:

(11) $$G_{o}(s)=1/(C_{2}s)$$

*G*_VR_(*s*) is the volatge regulation transfer function, where the conventional PI controller is always used. *K*_P_ and *K*_I_ are the proportional and integral coefficients of the PI regulator, respectively. Thus, we could obtain:

(12) $$G_{\mathrm{VR}}(s)=K_{P}+K_{I}s$$

*G*_F_(*s*) represents the transfer function of the load feedforward and *G*_MS_(*s*) is the relationship between *i*_M_ and *i*_s_. *G*_C_(*s*) is the transfer function of the deadbeat current controller. Considering a one-cylce delay, it could be written as:

(13) $$G_{C}(s)=\frac{1-e^{-sT_{s}}}{s}$$

The feedforward transfer function *G*_F_(*s*) can be calculated using small-signal analysis based on Equation (10). To substitute $i_{o}={\bar{i}}_{o}+{\overset{\wedge }{i}}_{o}$ and $I_{M}={\bar{I}}_{M}+{\overset{\wedge }{I}}_{M}$ into (10), ignoring the higher-order terms, *G*_F_(*s*) be derived as:

(14) $$G_{F}(s)={\overset{\wedge }{I}}_{M,F}(s)/{\overset{\wedge }{i}}_{o}(s)=1/(N(1-4fLkI_{M}/V_{1}))$$

The average output current of H bridge in the secondary side is derived as:

(15) $$i_{s}=\frac{V_{1}N}{2fL}D(1-D)$$

Combing (1) with (16), the *G*_MS_(*s*) could be derived as:

(16) $$G_{\mathrm{MS}}(s)={\overset{\wedge }{i}}_{S}(s)/{\overset{\wedge }{I}}_{M}(s)=N(1-4fLkI_{M}/V_{1})$$

According to the small signal model in Figure 5, the output impedance *R*_o1_(*s*) without and with feedforward could be calculated as (17) and (18), respectively.

(17) $$R_{o1}(s)=\frac{{\overset{\wedge }{V}}_{2}(s)}{{\overset{\wedge }{i}}_{o}(s)}=-\frac{G_{o}(s)}{1+G_{\mathrm{VR}}(s)G_{C}(s)G_{\mathrm{MS}}(s)G_{o}(s)}$$

(18) $$R_{o2}(s)=\frac{{\overset{\wedge }{V}}_{2}(s)}{{\overset{\wedge }{i}}_{o}(s)}=\frac{\left(G_{F}(s)G_{C}(s)G_{\mathrm{MS}}(s)-1)G_{o}(s\right)}{1+G_{\mathrm{VR}}(s)G_{C}(s)G_{\mathrm{MS}}(s)G_{o}(s)}$$

**Figure 6:**
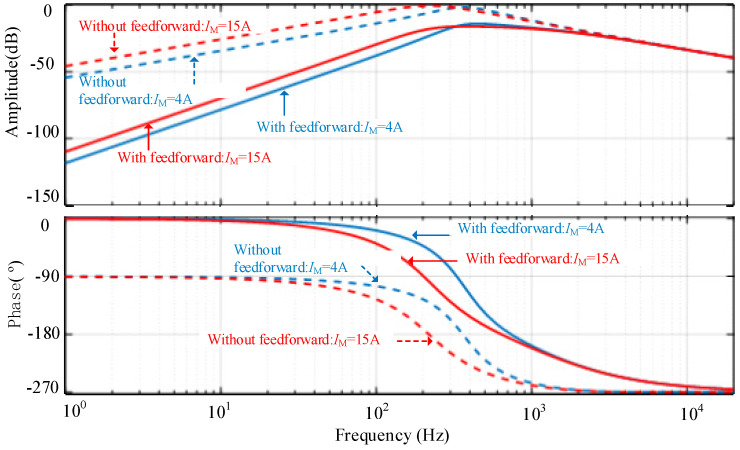
Output impedance of the converter with different loads.

By substituting the circuit parameters and control parameters into Equations (17) and (18), baud diagrams of output impedance with different loads under the double-closed-loop control strategy can be drawn as shown in Figure 6. In this case, the inductance *L =* μH, the voltage *V*_1_ = 300V and *V*_2_ = 280V.The coefficients of the PI regulator are *K*_P_ = 2 and *K*_I_ = 4000.

As shown in Figure 6, the closed-loop output impedance at low frequency decreases significantly after the feedforward is added. When *I*_M_ = 4 A, the output impedance at a frequency of 100 Hz decreases from −15 dB to −35 dB, whereas when *I*_M_ = 15 A, the output impedance decreases from −8 dB to −30 dB at 100 Hz. If the frequency is further reduced, the amplitude attenuation of the closed-loop output impedance brought by the feedforward control become more obvious, which indicates a more robust output voltage under the load disturbance.

## 4. Experimental Verification

### 4.1. Experimental Platform

**Figure 7:**
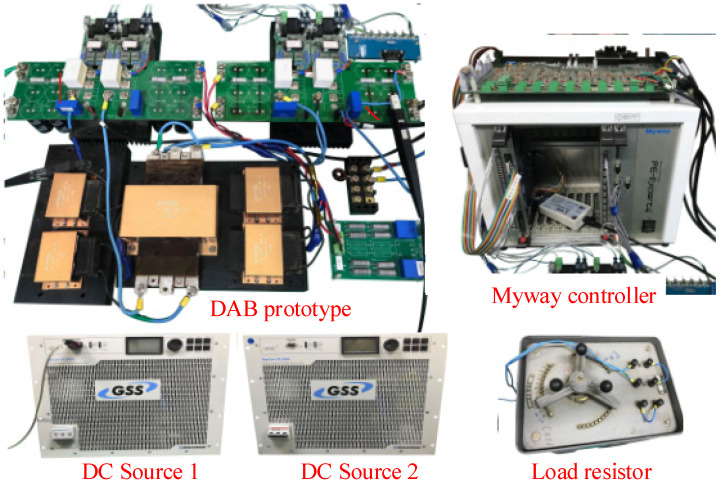
Experimental platform.

The laboratory IBDC experimental platform shown in Figure 7 was used to verify the proposed control method. The main circuit parameters are listed in Table 2. The current sensor LA55-P had a 200 kHz bandwidth from LEM. PE-Expert4 from Myway was utilized as the digital controller including DSP and FPGA cores. FPGA XC6SLX45 was used to generate PWM signals. The control variables were calculated in each cycle in DSP, and the corresponding PWM compare values CMP1 and CMP2 were updated at the beginning of the next cycle.

### 4.2. Comparisons of Different Current Controllers for Forward Power Transmission

The performance of the CSPS modulation-based current controller in [5] and the proposed ESPS-PCM were compared for the forward power transmission mode.

**Figure 8:**
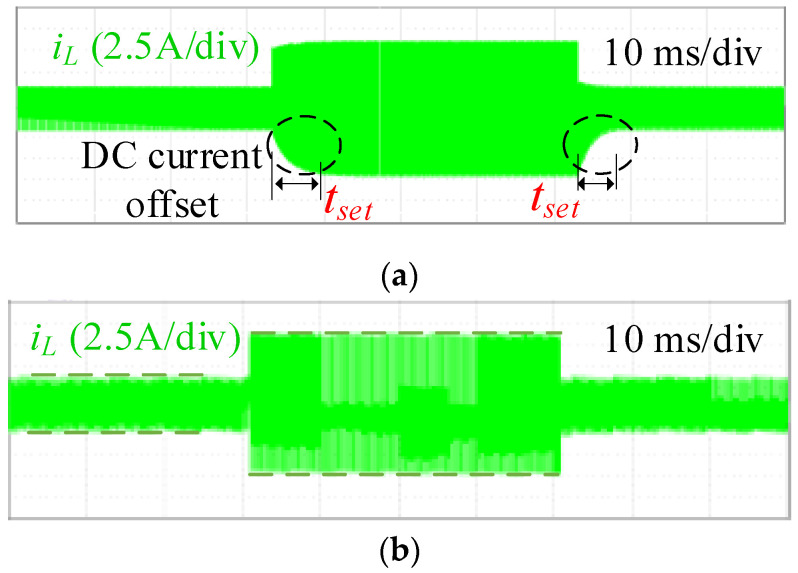
Zoomed-out waveforms during the step change of the current reference for forward power transmission. (**a**) CSPS modulation-based current controller. (**b**) ESPS-PCM controller.

Figure 8 shows the experimental waveforms of current *i*_L_ when the references have step changes. The references could be tracked for all the controllers when the current reference steps up from 3 A to 8 A and steps down from 8 A to 3 A. The settling time *t*_set_ in Figure 8a is obvious, while the settling time in Figure 8b is negligible. Additionally, the ESPS-PCM controller eliminates the transient DC current offset that exists in CSPS modulation-based controller, as shown in the dashed circle of Figure 8a.

**Figure 9:**
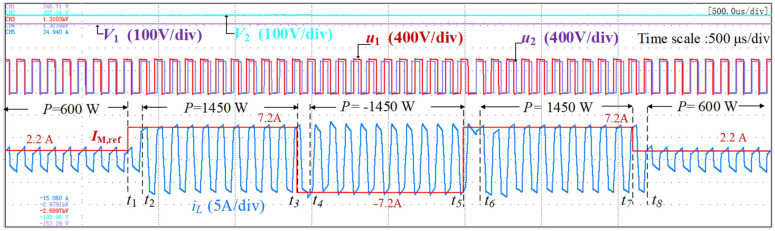
Transient waveforms of bidirectional power transmission.

To verify the performance of the current controllers during bidirectional power transmission, a sequence of current references was set to investigate the response. Figure 9 shows the waveforms of the ESPS-PCM controller. The transmission power *P* stepped up from 600 W to 1450 W at *t*_1_, changed direction to −1450 W at *t*_3_, reversed direction to 1450 W at *t*_5_ and then stepped down to 600 W at *t*_7_. The current references were smoothly reached with a one-cycle delay during the whole transient process, including the transition between two opposite power transmissions.

### 4.3. Dynamic Performance Comparison between Different Control Methods

**Figure 10:**
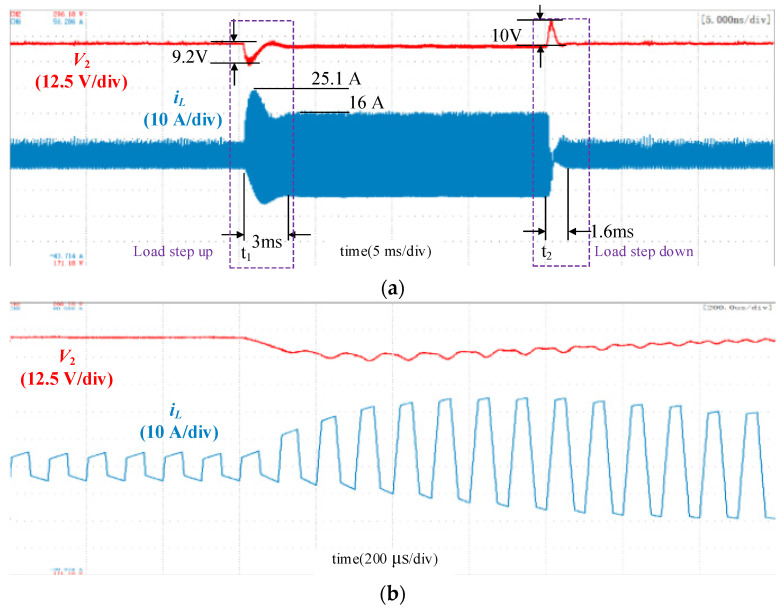

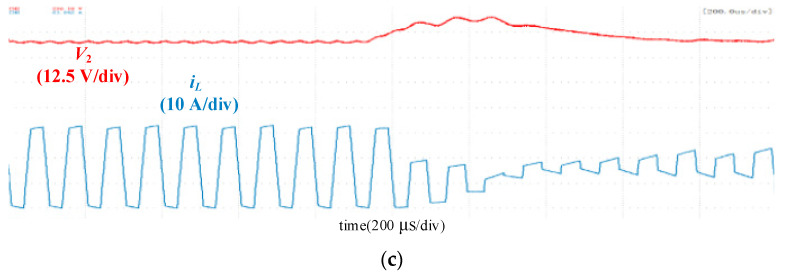
Waveforms using the conventional single-loop control. (**a**) Overall waveforms; (**b**) Zoomed-in waveforms during the load increase; (**c**) Zoomed-in waveforms during the load decrease.

**Figure 11:**
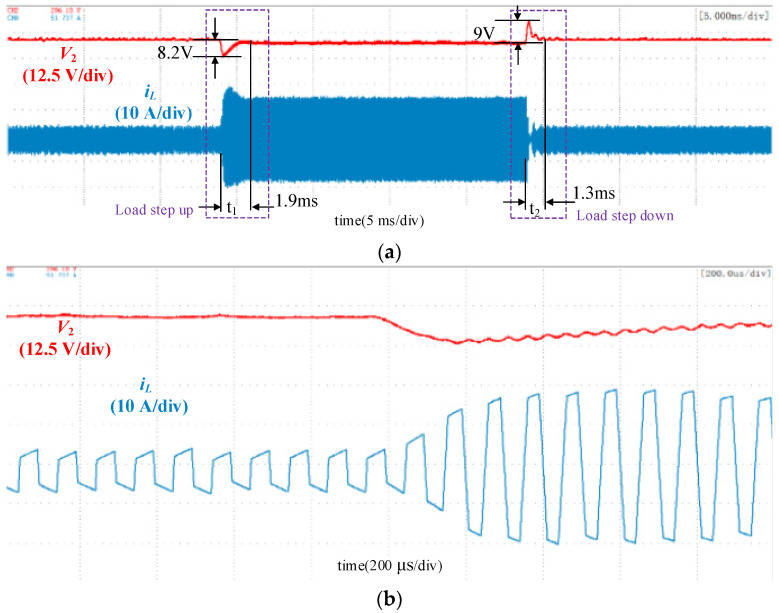

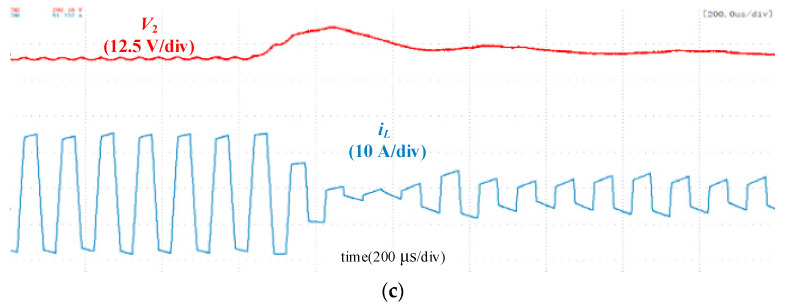
Waveforms using the double-closed-loop control. (**a**) Overall waveforms; (**b**) Zoomed-in waveforms during the load increase; (**c**) Zoomed-in waveforms during the load decrease.

**Figure 12:**
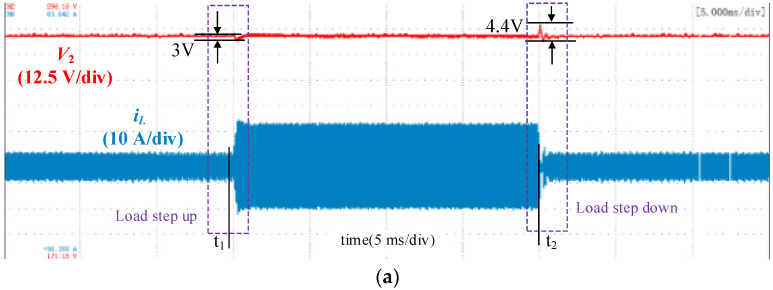

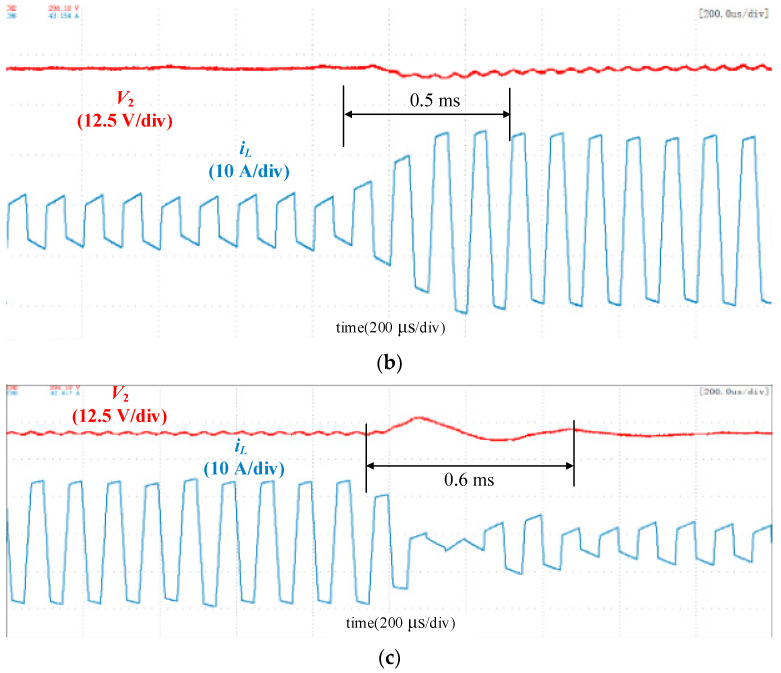
Waveforms using the double-closed-loop control with load feedforward. (**a**) Overall waveforms; (**b**) Zoomed-in waveforms during the load increase; (**c**) Zoomed-in waveforms during the load decrease.

The experimetal results with traditional single-voltage loop control, double-closed-loop control and double-closed-loop control with feedforward under load disturbance are shown in Figure 10, Figure 11 and Figure 12. The output voltage *V*_2_ was 280 V, and the load increased abruptly at *t*_1_ with load resistance decreases from 75 Ω to 25 Ω and decreased sharply at *t*_2_ with load resistance increases from 25 Ω to 75 Ω. When the load increases, the DC voltage will fall, and the control loop will increase the phase shift angle to transfer more power to maintain the DC load. Simillarly, when the DC load decreases, the DC voltage will rise and the control loop will decrease the phase shift angle to reduce the transmitted power.

Figure 10 shows the waveform using the conventional single-loop control. The inductor current shows obvious transient DC bias during abrupt load change. When the load increased, the peak current reached 25.1 A, and the current overshoot was 9.1 A. In the experiment, both the static and dynamic performances under load increase and decrease were considered when tuning the PI parameters. The waveform showed that the voltage sag was 9.2 V, and the settling time was 3 ms when the load increased. The voltage overshoot was 10 V, and the settling time was 1.6 ms when the load decreased. Figure 11 shows the waveform using the double-closed-loop control. The inductor current was symmetrical, and the transient DC bias was eliminated. When the load increased, the voltage sag was 8.2 V, and the settling time was 1.9 ms. The voltage overshoot was 9 V, and the settling time was 1.3 ms when the load decreased.

Figure 12 shows the waveforms using the double-closed-loop control with load feedforward. The transient DC bias was eliminated. During the transient process, the DC voltage variation was significantly reduced, and the settling time was obviously shortened compared with the double-closed-loop control without load feedforward. The voltage sag was 3 V when the load increased, and the voltage overshoot was 4.4 V when the load decreased. In the process of load surge, the recovery time for DC voltage was 0.5 ms, which is five switching cycles. The recovery time was 0.6 ms, which is six switching cycles, in the process of load decreases.

Compared with the traditional single-voltage loop control, the double-closed-loop control utilizes the deadbeat current controller as the inner loop, which directly regulates the high-frequency AC current of the transformer. Thus, the transient DC current bias could be eliminated. Meanwhile, the dynamic performance the of the IBDC with voltage mode mainly depends on the bandwidth of the feedback signal [12]. With the feedforward control samples, the load changes directly, which could significantly increase the robutness of the DC voltage under the load disturblances. The dynamic performance enhancement of the proposed control can be seen by comparison of Figure 11 and Figure 12.

## 5. Conclusions

A double-closed-loop control strategy based on the deadbeat current controller was proposed in this paper, which directly regulates the high-frequency inductor current to the reference and eliminates the transient DC current bias during the transient process. Furthermore, load feedforward was introduced to enhance the dynamic of the converter. The proposed control method shows potential in the application of IBDC under voltage mode. With the proposed method, the settling time could be reduced to within several PWM cycles during load disturbance. A 5 kW IBDC converter prototype was built, and the superior dynamic performance of the proposed control strategy was verified by the experimental results.

## Figures and Tables

**Table 1:** Control variable calculation with one-cycle delay compensation.

Mode		$t_{D}(n)$	$t_{W}(n)$
CSPS $(P_{\mathrm{ref}}\;\geq \;$0)		$\frac{kL(I_{M,\mathrm{ref}}(n)-I_{M}(n-1))}{2V_{1}}+t_{D}(n-1)$	$\frac{1}{2f}$
ESPS-PCM	Case 1	$\frac{kL(I_{M,\mathrm{ref}}(n)-I_{M}(n-1))}{2V_{1}}+t_{D}(n-1)+t_{W}(n-1)-\frac{1}{2f}$	$\frac{kL(I_{P2,\mathrm{ref}}(n)+I_{P2}(n-1))}{2V_{1}}-(k-1)(t_{D}(n-1)+t_{W}(n-1))+\frac{6-k}{4f}$
	Case 2	$\frac{kL(I_{P2,\mathrm{ref}}(n)-I_{P2}(n-1))}{2V_{1}}+\frac{1}{4f}$	$\frac{kL(I_{P2,\mathrm{ref}}(n)+I_{P2}(n-1))}{2V_{1}}-\frac{k-3}{4f}$
	Case 3	$\begin{array}{l} -\frac{kL(I_{P2,\mathrm{ref}}(n)+I_{P2}(n-1))}{2V_{1}}+(k-1)(t_{D}(n-1)\\ +t_{W}(n-1))+\frac{k-3}{4f} \end{array}$	$\begin{array}{l} -\frac{kL(I_{P2,\mathrm{ref}}(n)-I_{P2}(n-1))}{2V_{1}}-(k-1)(t_{D}(n-1)\\ +t_{W}(n-1))+\frac{5}{4f} \end{array}$
	Case 4	$-\frac{kL(I_{P2,\mathrm{ref}}(n)+I_{P2}(n-1))}{2V_{1}}+\frac{k}{4f}$	$-\frac{kL(I_{P2,\mathrm{ref}}(n)-I_{P2}(n-1))}{2V_{1}}+\frac{1}{4f}$
ESPS-MCM		$\frac{kL(I_{M,\mathrm{ref}}(n)-I_{M}(n-1))}{2V_{1}}+t_{D}(n-1)+t_{W}(n-1)-\frac{1}{2f}$	$\frac{kL(I_{M,\mathrm{ref}}(n)+I_{M}(n-1))}{2V_{1}}-t_{D}(n-1)-t_{W}(n-1)+\frac{5}{4f}$

**Table 2:** System Parameters.

Input voltage *V*_1_	300 V	Output voltage *V*_1_	280 V
Turns ratio *n*	1:1	Switching frequency *f*	10 kHz
Primary capacitor *C*_1_	2460 μF	Secondary capacitor *C*_2_	2460 μF
Inductor *L*	652 μH	Equivalent resistor *Rs*	80 mΩ

## Data Availability

Not applicable.
